# Predictive Parameters of Oral Health Quality of Life in Complete Mandibular Denture Wearers Stabilized by Mini-Implants: A Two-Year Follow-up Study

**DOI:** 10.3390/ma10101197

**Published:** 2017-10-19

**Authors:** Cindy Batisse, Guillaume Bonnet, Jean-Luc Veyrune, Emmanuel Nicolas, Marion Bessadet

**Affiliations:** Université Clermont Auvergne, CROC EA4847, BP 10448, F-63000 Clermont-Ferrand, France; CHU de Clermont-Ferrand, Service d’Odontologie, F-63000 Clermont-Ferrand, France; cindy.lance@uca.fr (C.B.); guillaume.bonnet@uca.fr (G.B.); j-luc.veyrune@uca.fr (J.-L.V.); marion.bessadet@uca.fr (M.B.)

**Keywords:** mini-implants, complete denture, GOHAI, quality of life

## Abstract

The frequent instability of mandibular removable complete dentures affects patient Oral Health Related Quality of Life (OHRQoL). An innovative therapeutic strategy used to improve stability involves placing four symphyseal mini-implants. This study was aimed at assessing OHRQoL over time in subjects in which mini-implants were placed and exploring if certain parameters could predict the evolution of their OHRQoL. The Quality of life of subjects with dentures was assessed using the Geriatric Oral Health Assessment Index (GOHAI) before (T0), 2–6 months (T1), twelve months (T2) and twenty-four or more months (T3) after mini-implant setting. Age, gender and chewing ability were tested as explanatory variables for the change in OHRQoL with time. Thirteen women and six men were included (mean age: 69 ± 10 years). After mini-implant placement, mean GOHAI scores at T1, T2 and T3 increased significantly (*p* < 0.001). The GOHAI-Add mean score was not affected by age or gender. Baseline chewing ability impacted the “functional” and “pain and discomfort” fields of the mean GOHAI scores (*p* < 0.05). The OHRQoL was quickly improved after mini-implant placement in complete denture wearers and then stabilized over time. Baseline chewing ability can be used as a predictive parameter of OHRQoL.

## 1. Introduction

The frequent lack of stability and retention of mandibular prosthesis in complete edentulous patients often results in dissatisfaction. Oral Health Related Quality of Life (OHRQoL) is particularly affected by discomfort and functional difficulties.

The placement of two symphyseal implants to support mandibular dentures (overdenture treatment) is recommended as “the first choice standard of care for edentulous patients” by the McGill consensus statement and more recently by the York consensus statement [1,2]. However, many geriatric edentulous patients have a variety of systemic diseases and frequently exhibit severe bone resorption that can make implant placement impossible. Furthermore, this treatment presents many disadvantages such as cost and an extended treatment period with delayed loading of the prosthesis [3,4]. 

An alternative treatment consists on the placement of four inter-foraminal mini-implants to stabilize the complete mandibular denture. The Glossary of Oral and Maxillo facial Implants (GOMI) has defined the term “mini implant” as an “implant fabricated with the same biocompatible materials as others implants but of smaller dimensions” [5]. Mini-implants allow reducing trauma for elderly patients and increase treatment possibilities. Using mini-implants offers many advantages such as implant placement in a narrow site, minimally invasive surgery, short treatment duration with immediate loading and cost-effective care [6]. However, the term “mini implants” may have a negative connotation and the alternative term “Narrow Diameter Implants” does not emphasize its specific use in geriatric patients. Therefore, the term “Geriatric Slim Implants” (GSI), as proposed previously [3], seems more accurate regarding their use in this procedure. Previous studies confirmed the reliability of GSI placement as a retentive element for mandibular complete dentures [7,8] in the medium term. Other studies showed that GSI placement had a positive impact on patients’ satisfaction, OHRQoL [9,10,11,12,13,14] and masticatory function [10,15]. However, the possible link between improved mastication after GSI placement and the evolution of OHRQoL has not yet been explored.

The aim of this study was, firstly, to assess changes in OHRQoL after stabilization of mandibular complete dentures with GSI in the short-term (2–6 months and 1 year after GSI placement) and in the medium term (2 years and more after GSI placement). Secondly, the study determined if parameters such as gender, age and chewing ability could be used to predict the evolution of patients’ OHRQoL.

## 2. Results

### 2.1. Study Sample

Nineteen subjects (thirteen women and six men) were included (mean age: 69 ± 10 years). For this study, ten of these subjects were evaluated on their chewing ability at T0 and T1. 

### 2.2. Influence of GSI Placement on Oral Health Related Quality of Life

**Figure 1:**
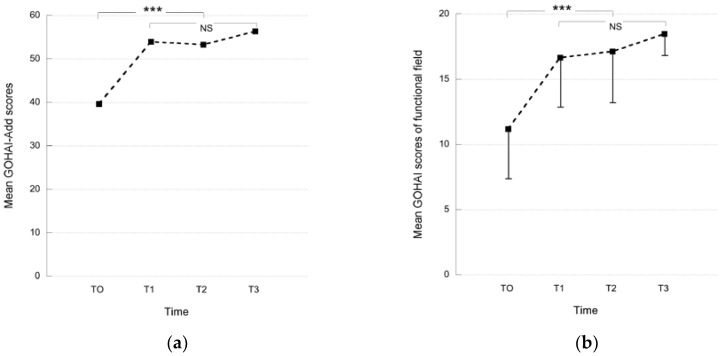

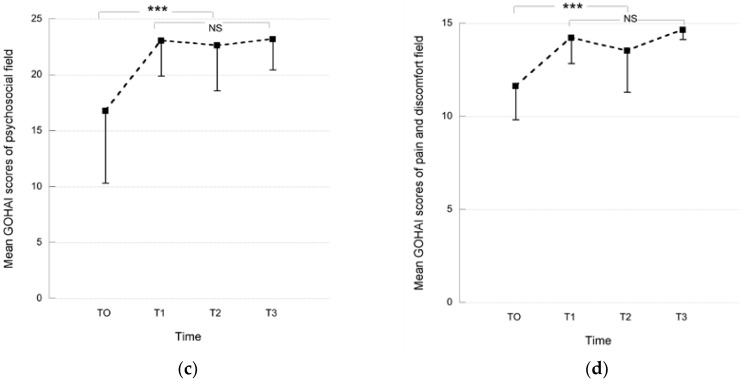
Mean GOHAI scores (SD) obtained before (T0) and after (T1: 2–6 months after GSI placement, T2: 12 months after GSI placement and T3: 24 months and more) treatment: (**a**) Mean GOHAI-Add scores; (**b**) Mean GOHAI scores of the “functional” field; (**c**) Mean GOHAI scores of the “psychosocial” field; (**d**) Mean GOHAI scores of the “pain and discomfort” field (NS: Non Significant; *** *p* < 0.001).

The mean GOHAI scores measured at T0 (before GSI placement), at T1 (2–6 months after GSI placement), at T2 (12 months after GSI placement) and T3 (24 months and more after GSI placement) are presented in Figure 1. After GSI placement, the mean GOHAI scores (T1, T2 and T3) were significantly higher than those of T0 for all the GOHAI fields (Post Anova Student Newmans Keuls test, *p* < 0.001: F = 13: Gohai-Add; F = 14: functional field; F = 9: psychosocial field; F = 10: pain and discomfort field) (Figure 1). At T0, nearly all the subjects had a poor OHRQoL. At T1, the OHRQoL of five subjects remained poor despite an increase of their GOHAI-Add score, and nine subjects had a GOHAI-Add score higher than 56, corresponding to a satisfactory OHRQoL. No significant difference was observed for the mean GOHAI score values when comparing the data obtained at T1, T2 and T3 (Figure 1).

#### 2.2.1. Influence of Socio-Demographic Criteria on Oral Health Related Quality of Life

Thirteen women and six men with a mean age of 69 years ± 10 (min 57 years, max 95 years) participated in this study. Nine subjects were 69 years of age or younger and ten were older than 69 years. Multivariate analysis showed that neither age nor gender had an influence on the GOHAI-add score at T0, T1 and T2.

#### 2.2.2. Influence of Chewing Ability on Oral Health Related Quality of Life

Before treatment (T0), five subjects out of ten were able to chew the carrot sample, while the five others were unable to do so. After treatment (T1), only 2 subjects refused to chew carrots. Thus, there were 2 subjects in the group unable to chew the raw carrot sample before and after treatment (“refusal group”); three subjects in the group able to chew the raw carrot sample only after treatment (“improvement group”) and five subjects in the group always able to chew the raw carrot sample (“acceptance group”).

The ability to chew the raw carrot sample at T0 had an impact on the evolution of the GOHAI “functional” field (F = 7, *p* < 0.05) and “pain and discomfort” field mean scores (F = 11, *p* < 0.01) from T0 until T2. But chewing ability had no influence on the evolution of the GOHAI-Add mean score or the “psychosocial” field mean score (T0 to T2).

Whether or not chewing ability evolved between T0 and T1, there was no impact on the GOHAI mean scores at the different times (T0, T1 and T2).

## 3. Discussion

The aim of this study was to assess whether stabilized mandibular complete denture with GSI improved patients’ OHRQoL and whether the OHRQoL changes were influenced by sociodemographic factors or by chewing ability. The main results of the study were: (1) quality of life related to oral health was quickly improved after GSI placement and then remained stable over time; (2) sociodemographic factors had no impact on OHRQoL changes; (3) the baseline chewing ability of edentulous subjects could be a predictive factor of the evolution of patients’ oral health related quality of life.

Certain limits of this study can be outlined. Firstly, all the available studies on OHRQoL with GSI have used the OHIP questionnaire [9,10,11,16], which is the most widely used OHRQoL instrument. In this study, the OHIP questionnaire was not chosen to assess quality of life because no French version was validated. A recent study [17] proposed to integrate items of OHRQoL instruments such as the GOHAI and the OHIP in a four dimension OHRQoL model consisting of Oral Function, Orofacial Pain, Orofacial Appearance, and Psychosocial Impact. In the future, this model could facilitate comparisons between studies.

Secondly, the study has a small sample number. GSI placement is indicated only in specific cases, in elderly patients who cannot received symphyseal implants for anatomical, medical or economic reasons. For all others patients, the mandibular complete denture can be stabilized with two symphyseal implants. Thus, the number of patients who received GSI treatment is restricted. Also, long term follow-up can be more difficult in these elderly patients.

The stabilization of mandibular complete dentures by GSI tended to improve the patients’ OHRQoL. All the GOHAI fields were improved by GSI treatment. Before GSI placement, the mean GOHAI-Add score was 39 (±11), reflecting a poor OHRQoL, although after GSI placement the mean GOHAI-Add score increased to 54 (±8). At T1, nine subjects had a mean GOHAI-Add score higher than 56, corresponding to a satisfactory OHRQoL. Only five subjects conserved a poor OHRQoL, although their mean GOHAI-Add score increased. Previous studies showed that the placement of GSI improves patient satisfaction and oral quality of life [9,10,11,12,13,14,18]. An increase in OHRQoL was also demonstrated for mandibular implant overdentures [19,20,21]. These studies confirmed that stabilization and retention are major factors contributing to the success of removable dental prosthesis treatment, and are partly responsible for patients’ poor OHRQoL and satisfaction. In patients with implant therapy limitations, GSI treatment provides an alternative therapeutic solution to prostheses remake. A study has shown that oral health status was significantly better after implant overdenture treatment than after new conventional denture treatment [21]. Indeed, renewing removable dentures only moderately improved oral health related quality of life [22]. 

No significant difference was observed in the mean GOHAI scores (GOHAI-Add and field) between each time after GSI treatment. After 1 year of treatment, OHRQoL became stable. Improvement of OHRQoL occurred in the first few months after GSI placement. Other authors observed the same results [10]. All these results suggest that the quality of life of edentulous persons improved before stabilizing, whether mini-implant or conventional implant treatment was applied [9,10,19,21,23,24]. A study reported that the effect of mandibular two-implant overdentures on OHRQoL is stable over a 2-year period [25]. A long-term study is necessary to confirm the stabilization of OHRQoL over time with GSI treatment. However, no matter which implant treatment was applied, the OHRQoL obtained was never able to reach that observed in normal dentate patients.

Changes in the OHRQoL of patients wearing GSI supported mandibular overdenture were not influenced by sociodemographic parameters such as gender and age. These results confirmed previous data also obtained with narrow diameter implants in edentulous persons [10]. In contrast, baseline chewing ability had an influence on the evolution (T0 to T2) of the “functional” and “pain and discomfort” fields of the mean GOHAI scores (respectively, *p* < 0.05 and *p* < 0.01). Although many other factors could have an impact on OHRQoL, baseline chewing ability seemed to be essential. Indeed, edentulous subjects and denture wearers frequently report daily chewing difficulties [26,27]. These difficulties affect their eating habits, social interactions and comfort [28,29] and thus their OHRQoL. Baseline chewing ability could be used as a predictive parameter of changes of OHRQoL in complete denture wearers with GSIs for mandibular overdentures. In this study, no link was observed between changes in chewing ability and changes in GOHAI scores. This could be explained by the limited number of subjects included and would require additional inclusions. 

The authors showed that evaluating the case severity of edentulousness with an index [30] or with prognostic indicators [31] before treatment allowed predicting clinical outcomes when fabricating new complete dentures. Similarly, according to the results of this study, when implant treatment is considered, a chewing test could be implemented to predict the evolution of patients’ OHRQoL. The usual chewing test with model or natural test foods could be employed, but these require materials (test food, camera or electromyography), time and the results that are not immediately available. A color-changeable chewing gum could be used [10,32]. This chewing test has the advantages of being quick and easy to use for clinical purposes. This chewing-gum test could become a diagnostic tool during the first medical consultation in the same way as a radiographic examination.

This study confirmed that GSI placement had a positive impact on OHRQoL. Other studies on mini-implant survival reported satisfactory medium term success rates [7,9,33]. A long-term study is therefore necessary to confirm the reliability of GSI placement. Independently, prosthetic maintenance nonetheless remains essential [8,13]. This alternative mini-implant procedure could be an advantageous treatment option for geriatric patients unable to receive conventional implants. However, even though OHRQoL improved quickly after GSI placement, another study reported that masticatory function remained deficient compared to that of dentate persons [15]. These results highlighted the limited adaptation capacity of geriatric people. Indeed, adaptability to new or changed situations tends to decrease with age.

## 4. Materials and Methods

### 4.1. Study Design

The prospective observational study was conducted at the Dental Department of the University Hospital of Clermont-Ferrand, France over a 5-year period (2012–2017), and was approved by the local Ethics Committee (CECIC- GREN-06-12).

### 4.2. Study Sample

Edentulous patients received new maxillary and mandibular complete dentures during the 6 months prior to this study. All the new dentures, anatomical posterior teeth with a 20° angle cusp manufactured by Ivoclar^®^ (SR Orthotyp PE, Schaan, Liechtenstein), were used with the bilateral balanced occlusion scheme. GSI treatment was proposed when (1) patients reported oral function difficulties with their mandibular denture in spite of a prosthetic adaptation period; (2) practitioners noted a lack of stability and did not have any technical solution to improve retention; and (3) “normal” implant treatment was not possible”. Mini-implant rehabilitation consisted of the placement of four symphyseal GSIs using a flapless procedure, followed by immediate loading of the GSIs with the O-ring prosthesis attachment. GSI is a one piece mini implant 2.7 mm in diameter and 9–15 mm long made of grade V titanium from Eurotecknica^®^ (Sallanches, France). The entire clinical protocol was previously described by Huard et al. [3]. The initial complete denture became an implant-supported overdenture. 

Every patient that underwent GSI treatment at the Dental Department of the University Hospital of Clermont-Ferrand, France from January 2012–December 2016 was included in the study. The following inclusion criteria were used: Cooperating adult patient, able to understand and participate in the study, wearing new, adapted complete dentures with an unstable mandibular denture, and for whom rehabilitation with “standard implants” was not possible due to medical, anatomical or/and economic reasons. Patients who could not speak or read French fluently, with cognitive disability according to their medical questionnaire, chronic disease which could disturb the healing process, smokers of more than ten cigarettes a day and with no social security were excluded.

At the end of the follow-up period (December 2016), some subjects were excluded for medical (death or deterioration of general health status) or mobility reasons. Out of the twenty-two subjects initially included, a complete dataset for nineteen participants was finally used for the study until T2 and, so far, eleven until T3.

The required sample size was estimated from a preliminary pilot study that measured the GOHAI score before rehabilitation (T0) and one year after (T2) (eight first included subjects, to January 2012 for April 2015). The mean GOHAI values increased from 33 to 50.37 respectively (SD = 12.15). Calculations were based on this difference for a continuous criterion with paired values and indicated the need at least for 18 subjects (α = 5%, β = 10%, epiR package 0.9–30).

### 4.3. Experimental Design

**Figure 2:**
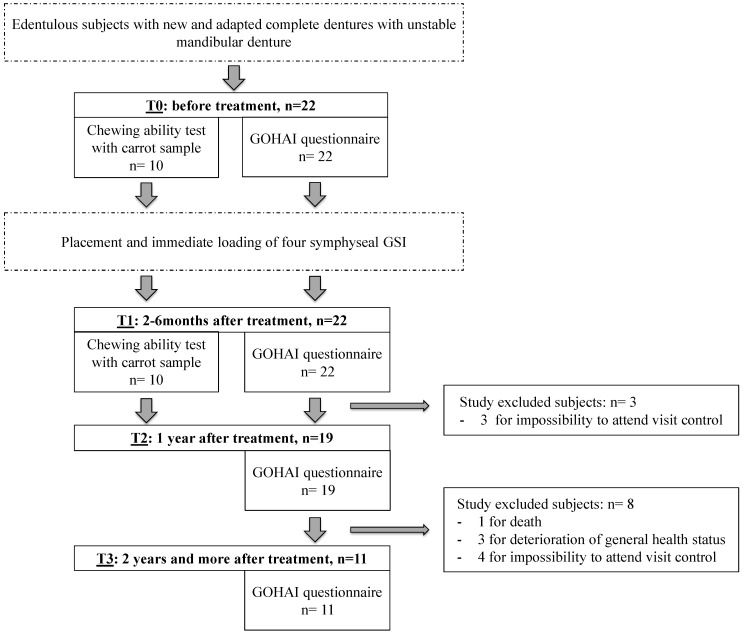
Experimental procedure. For statistical analysis, data on nineteen subjects were used to have a complete database from T0 to T2. Consequently, number of subject at each time was standardized.

Each subject answered the GOHAI questionnaire at T0 (before GSI placement), at T1 (two to six months after GSI placement), at T2 (1 year after GSI placement) and at T3 (2 years and more after treatment). At T0 and T1, they were asked to chew a sample of raw carrot to assess their chewing ability. Chewing ability at T0 and T1 could be evaluated for ten out of the nineteen subjects included (Figure 2). The flexible time-lapse of two or six months after GSI placement used at T1 was implemented to allow most of the subjects to come to the follow-up visit. 

### 4.4. Assessment Tools

#### 4.4.1. Oral Health Related Quality of life

The French version of the GOHAI questionnaire was used to assess the subjects’ OHRQoL [34]. This questionnaire provides a score based on the answers to twelve items grouped in three fields: The functional field (items 1–4), the psychosocial field (items 6, 7, 9, 10 and 11 concern relational discomfort and appearance), and the pain or discomfort field (items 5, 8 and 12 concern drugs, gingival sensitivity, and discomfort when chewing certain foods). A summary score (GOHAI-Add score) for each item (1 = always to 5 = never) was calculated. 

The maximum GOHAI score is 60 (20 = functional field; 25 = psychosocial field; 15 = pain or discomfort field). In this study, however, the subjects did not reply to the 12th item relating to dental sensitivity to heat and cold because they were edentulous. The maximum score of 5 was therefore attributed to each subject for this item. According to Atchison and al. [35], a score between 57 and 60 is regarded as high and corresponds to a satisfactory OHRQoL. A score from 51 to 56 is regarded as average, and a score below 50 is regarded as low, reflecting a poor OHRQoL.

#### 4.4.2. Chewing Ability

To test the subjects’ chewing ability, a sample of raw carrot was used. Carrots were bought fresh at a local market and cut into cylindrical samples (2 cm diameter; weight 4 g ± 0.05) just before the experiment. Food and sample dimension were chosen according to an ongoing study of a prospective follow-up evaluation of the impact of GSI retained mandibular denture on the masticatory function [15]. Each subject put one sample of raw carrot in their mouth and tried to chew on it. If they were unable to chew the proposed food, “food refusal” was reported. On the contrary, “food acceptance” was noted when the subjects were able to chew. Chewing ability was explored before (T0) and after GSI placement (T1). The subjects were then categorized into three groups: (i) “refusal group” when subjects were unable to chew the carrot sample at T0 and T1; (ii)”improvement group” when the subjects were unable to chew the carrot sample at T0 but managed to chew it at T1; and (iii) “acceptance group” when the subjects were able to chew the carrot sample at T0 and T1.

#### 4.4.3. Statistical Analysis

Statistical analysis was performed using SPSS^®^ (IBM, v20, New York, NY, USA) software. Statistical significance was set at *p* < 0.05. After checking for the normal distribution of the data, parametric tests were used to obtain the results.

The mean scores of each component of the GOHAI (GOHAI-add, “functional” field, “psychosocial” field and “pain or discomfort” field) were compared before (T0), 2–6 months after treatment (T1), 1 year after treatment (T2) and 24 months and more after treatment (T3) by ANOVA test, followed by a post-hoc Student-Newmans-Keuls test (α = 0.001). 

Furthermore, age (<70 years vs. >70 years), gender were tested as explanatory variables for the change in the mean GOHAI-add scores between the different times, assessed by multivariate analysis, and repeated measures procedures (dependent factors: GOHAI-add or each components of GOHAI, fixed factor: age; gender). To assess the relationship between chewing ability and GOHAI score, the analysis was only performed for the ten subjects that underwent the chewing ability test.

## 5. Conclusions

The quality of life related to oral health was quickly improved after GSI placement and then remained stable over time. Baseline chewing ability could be employed as a predictive parameter of changes in oral health quality of life in complete denture wearers when GSI is used to stabilize mandibular overdentures.
